# Urinary pH reflects dietary acid load in patients with type 2 diabetes

**DOI:** 10.3164/jcbn.16-118

**Published:** 2017-07-01

**Authors:** Akane Miki, Yoshitaka Hashimoto, Muhei Tanaka, Yukiko Kobayashi, Sayori Wada, Masashi Kuwahata, Yasuhiro Kido, Masahiro Yamazaki, Michiaki Fukui

**Affiliations:** 1 Department of Endocrinology and Metabolism, Kyoto Prefectural University of Medicine, Graduate School of Medical Science, 465 Kajii-cho, Kawaramachi-Hirokoji, Kamigyo-ku, Kyoto 602-8566, Japan; 2 Graduate School of Life and Environmental Sciences, Kyoto Prefectural University, 1-5 Shimogamo Hangicho, Sakyo-ku Kyoto-shi, Kyoto 606-0823, Japan

**Keywords:** type 2 diabetes, urinalysis, dietary assessment, diet, dietary acid load

## Abstract

Dietary acid load is important information, however, survey of food intake needs time and skill. Therefore, it is difficult to survey food intake from all patients. It remains to be elucidated the association between dietary acid load and urinary pH in patients with type 2 diabetes. In this cross-sectional study of 173 patients, we investigated the relationship between urinary pH and dietary acid load, assessed with potential renal acid load. Habitual food and nutrient intake was assessed by a self-administered diet history questionnaire. Urinary pH was negatively correlated with potential renal acid load (*r* = –0.24, *p* = 0.002). Multivariate regression analysis revealed that potential renal acid load (standardized regression coefficient = –0.21, *p* = 0.036) was associated with urinary pH after adjusting for covariates. In addition, according to the receiver operator characteristic analysis, the optimal cut-off point of urinary pH for high dietary acid load, defined as potential renal acid load over 7.0 mEq/day was 5.7 (area under the receiver operator characteristic curve 0.63 (95% CI 0.54–0.71), sensitivity = 0.56, specificity = 0.70, *p* = 0.004). Urinary pH was associated with dietary acid load in patients with type 2 diabetes. We suggest that urinary pH can be a practical screening marker for dietary acid load in patients with type 2 diabetes.

## Introduction

A food intake can affect the body’s acid balance via supply of acid or base precursors.^(1,2)^ It has been reported that, meat, fish, cheese, grain products or rice is a relatively strong net acidifying food and fruit or vegetables is a relatively strong net alkalinizing food.^(3)^

Recent studies revealed that a dietary acid load was a risk factor for hypertension^(4)^ and type 2 diabetes in general population.^(5,6)^ In addition, the dietary acid load is associated with a prevalence of metabolic syndrome in patients with type 2 diabetes.^(7)^ Therefore, the dietary acid load is an important information for not only general population but also patients with type 2 diabetes. However, it is difficult to survey food intake from all patients, because it takes too much time and exceptional skill to survey food intake.^(8)^

Previous studies reveled that urinary pH has a close association with dietary acid load in general population.^(9,10)^ Furthermore, previous studies also revealed that the more acidifying food is consumed, the more excess acid content is produced, which caused light metabolic acidosis in general population.^(11)^ In contrast, the pH of interstitial fluids, which is associated with metabolism of acid-base equilibrium,^(12)^ in diabetes mellitus is lower than that in non-diabetic control.^(13)^ In addition, metabolic acidosis is associated with development of insulin resistance and type 2 diabetes.^(14)^ Moreover, urinary pH values of patients with diabetes are lower than that of a general population.^(15)^ Therefore, there is a possibility that the metabolism of acid-base equilibrium of patients with type 2 diabetes was difference from that of general population. Thus, the purpose of this post-hoc analysis of cross-sectional study was to reveal the association between urinary pH and dietary acid load in patients with type 2 diabetes.

## Materials and Methods

### Study patients

We accessed a database of a previously reported study^(16)^ to evaluate the association between urinary pH and dietary acid load. We included type 2 diabetes patients who performed a self-administered diet history questionnaire (DHQ). We excluded the patients who did not complete the DHQ, who had chronic renal failure, defined as estimated glomerular filtration rate (eGFR) <30 ml/min/1.73 m^2^,^(17)^ thyroid disease and turner syndrome. Participants did not take potassium citrate which affects urinary pH.^(18)^ Approval for the study was obtained from the local research ethics committee of Kyoto Prefectural University of Medicine (Kyoto, Japan) and written informed consent was obtained from all patients.

### Data collection

Body mass index (BMI) was calculated as weight in kilograms divided by the square of height in meters. Hemoglobin A1c was assayed using high-performance liquid chromatography and was expressed as a NGSP unit. GFR was estimated using the Japanese Society of Nephrology equation: eGFR (ml/min/1.73 m^2^) = 194 × sCr^−1.094^ × age^−0.287^ (×0.739 for women).^(19)^ Urinary albumin and creatinine concentrations and urinary pH were determined using early morning spot urine. Urinary pH was measured by the indicator method using a dipstick and the available range was from pH 5.0 to pH 9.0 in 0.5 pH unit increments. A mean value for urine albumin excretion and urinary pH were determined from three urine collections.

### Estimation and assessment of habitual food and nutrient intake

The detail of estimation and assessment of habitual food and nutrient intake was reported previously.^(16)^ Briefly, we checked the usual dietary habits of the patients during a one-month period by DHQ.^(20)^ The questionnaire of DHQ was composed of questions regarding general dietary behavior, major cooking methods, the quantity and frequency of consuming 149 selected food and beverage items, and the sum of rice and miso soup consumed daily. The food and beverage items and portion sizes of DHQ were comes from the data in the National Nutrition Survey of Japan (NNSJ)^(16)^ and several recipe books for Japanese dishes. We calculated dietary total energy intake, carbohydrate intake, protein intake, fat intake, phosphorus intake, potassium intake, calcium intake, magnesium intake and fruit and vegetable intake, using DHQ and the nutritional value calculation program in this study.

### Assessment of dietary acid load scores

We used potential renal acid load (PRAL) as the parameter of dietary acid load score. The PRAL score suggests the intestinal absorption rates of contributing nutrient ionic balances for protein, potassium, calcium and magnesium and the disassociation of phosphate at pH 7.4.^(9)^ The PRAL (mEq/day) was defined as below^(1)^: PRAL (mEq/day) = 0.49 × protein (g/day) + 0.037 × phosphorus (mg/day) – 0.021 × potassium (mg/day) – 0.026 × magnesium (mg/day) – 0.013 × calcium (mg/day).

### Statistical analysis

We used JMP version 12.0 software (SAS Institute Inc., Cary, NC) for the statistical analyses and statistically signiﬁcant was defined as *p* value less than 0.05. We calculated mean or frequencies of potential confounding variables and data were expressed as mean ± SD and absolute number. We performed logarithmic transformation before performance of correlation and multiple logistic regression analyses, because triglycerides was a skewed variable. We used Spearman’s rank correlation coefficient to investigated the relationship between urinary pH and PRAL or the other variables was examined by multiple regression analysis was performed to examine the effects of various factors on urinary pH and the following factors were considered simultaneously as independent variables: age, sex, BMI, creatinine, uric acid, HbA1c, logarithm of triglycerides, fruit and vegetable intake, and PRAL. In addition, we also performed receiver operator characteristic (ROC) analysis to calculate area under the ROC curve (AUC) of urinary pH for high dietary acid load, defined as PRAL over 7.0 mEq/day.^(5,7)^

## Results

In this study, we enrolled 247 patients with type 2 diabetes (134 males and 113 females) who received DHQ. Among them, 202 patients (106 males and 96 females) completed the questionnaire. Then, we excluded 29 patients; incomplete information (11 males and 6 females), thyroid disease (1 male and 3 females), chronic renal failure (3 males and 2 females) and Turner syndrome patient (1 female), alcoholism (1 male), adrenal tumor (1 male). Finally, a total of 173 patients (89 males and 84 females) met the inclusion criteria.

Clinical characteristics of 173 patients with type 2 diabetes are shown in Table 1. The average age, HbA1c and urinary pH of the patients were 65.0 years old, 7.3% (56 mmol/mol) and 6.0, respectively. In addition, the average PRAL was 5.8 mEq/day.

Relationship between urinary pH and PRAL or the other variables is shown in Table 2. Urinary pH was negatively correlated with creatinine (*r* = –0.18, *p* = 0.016), uric acid (*r* = –0.16, *p* = 0.041), PRAL score (*r* = –0.24, *p* = 0.002). In addition, urinary pH was positively correlated with fruit and vegetable intake (*r* = 0.15, *p* = 0.045).

**Fig. 1:**
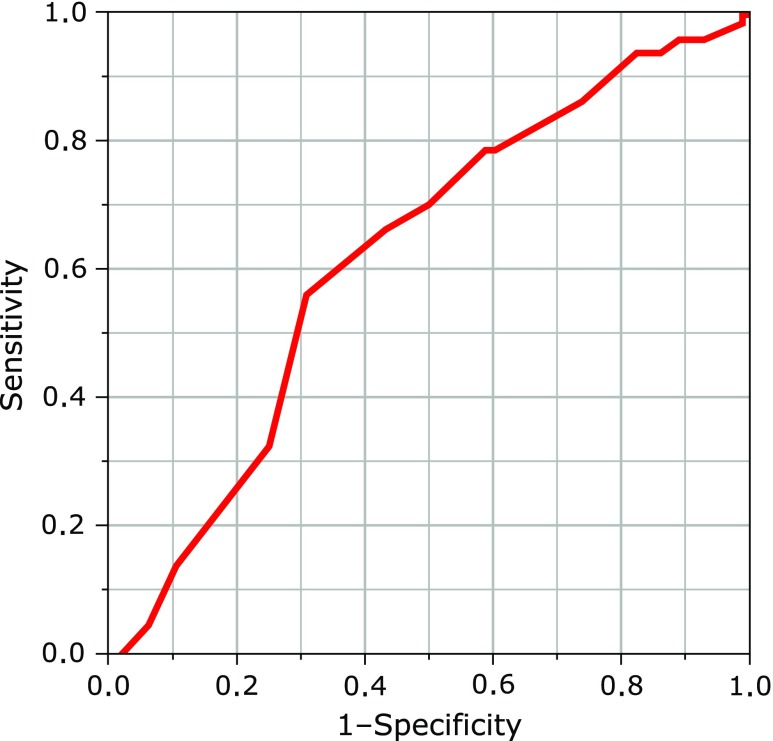
Receiver operating characteristic (ROC) curve and area under the ROC curve (AUC) showing the ability of urinary pH for higher dietary acid, which was defined as PARL over 7.0 mEq/day. The optimal cut-off point of urinary pH for higher dietary acid, defined as PARL over 7.0 mEq/day was 5.7 [AUC 0.63 (95% CI 0.54–0.71), sensitivity = 0.56, specificity = 0.70, *p* = 0.004].

Multiple regression analysis on urinary pH is shown in Table 3. PRAL score was independently correlated with urinary pH (standardized regression coefficient = –0.21, *p* = 0.036). According to the ROC analysis (Fig. 1), the optimal cut-off points of urinary pH for high dietary acid load were 5.7 (AUC 0.63 (95% CI 0.54–0.71), sensitivity = 0.56, specificity = 0.70, *p* = 0.004) for PRAL over 7.0 mEq/day.

## Discussion

The major finding of this study is that there is an association between urinary pH and dietary acid load in patients with type 2 diabetes. The information of dietary acid load is important for general population, because high dietary acid load is a risk of hypertension,^(4)^ type 2 diabetes^(5,6)^ and chronic kidney disease.^(21)^ Additionally, the information of dietary acid load is also important for patients with type 2 diabetes, because higher dietary acid load is associated with metabolic syndrome in patients with type 2 diabetes.^(7)^ However, it is difficult to survey food intake from all patients, because it takes too much time and skill to survey food intake.^(8)^ Previous studies revealed that there is an association between urinary pH and dietary acid load in general population.^(9,10)^ We revealed for the first time the association between urinary pH and dietary acid load in patients with type 2 diabetes.

Acidogenic food can induce an acid load and this can cause chronic metabolic acidosis.^(1)^ Renal excretion of H^+^ and regeneration of HCO_3_^−^ is one of the main mechanisms of maintaining the acid equilibrium in the body.^(12,22)^ In fact, increased endogenous acid production leads to increased renal net acid excretion in both animal^(23)^ and human^(24)^ models. Therefore, the dietary acid load is associated with urinary pH and net acid excretion.^(22,23,25)^ Previous studies revealed that urinary pH values of patients with diabetes are lower than controls matching by BMI^(15)^ and the lower pH is due to a combination of lower ammonia excretion and greater net acid excretion. However, one of the causes of greater net acid excretion in patients with type 2 diabetes might be also associated with lower intake of dietary alkali and greater intake of dietary acid.^(26)^

In this study, we also revealed the optimal cut-off point of urinary pH for high dietary acid load was 5.7. Thus, if we find the type 2 diabetic patients of low urinary pH (urinary pH <6.0), we should performed food intake survey and dietary intervention.

This study has some considerable limitations. First, we assessed the food intakes of the patients using the DHQ. Since all the dietary variables were obtained by the self-reported questionnaires, the accuracy of diet survey depend on memorial power of patients. However, DHQ was correlated with total energy expenditure by the doubly labeled water^(27)^ or semi-weighted dietary record,^(28)^ which is a standard method. Second, this was a single-site study and the sample size was relatively small. Thus, the study population might not accurately represent the underlying population. Third, a control population matched by BMI could be useful to demonstrate the difference trendlines of diabetes and non-diabetes populations. Fourth, there is a possibility that when the urine exposed to environment, pH turns alkaline because of loss of CO_2_, which gives a false impression, although we measured urinary pH as soon as possible. Lastly, we used single, not 24-h collected, urine to measure urinary pH and we also measured urinary pH by the indicator method using a dipstick rather than electrodes. However, we used average of three time collection urine. In addition, a previous study showed that fasting urinary pH correlates significantly with 24-h urinary pH.^(29)^ Moreover, a previous study also showed the accuracy of urinary dipstick testing.^(30)^ Furthermore, urinary pH changes along the day with a circadian rhythm with more alkaline peaks after meals and fasting morning pH reflects the effect of basal metabolism whereas daily pH is a more reliable estimate of total proton excretion including the component from diet. However, basal metabolism was also affected by dietary acid load.^(31)^ Therefore, fasting morning pH might also reflect total proton excretion including the component from dietary habits.

In conclusion, to the best of our knowledge, the present study is the first study of the relationship between urinary pH and dietary acid load in patients with type 2 diabetes. In addition, we also showed that the acidic urine (urinary pH = 5.0 or 5.5) is associated with higher dietary acid intake. We suggest that examination of urinary pH can be a practical screening tool for dietary acid load in patients with type 2 diabetes.

## Author Contributions

A.M. originated and designed the study, researched data and wrote manuscript. Y.H. originated and designed the study, researched data and reviewed the manuscript. M.T. designed the study, researched data and contributed to discussion. Y.K., S.W., M.K., Y.K. and M.Y. researched data and contributed to discussion. M.F. researched data and reviewed and edited the manuscript. M.F. is the guarantor of this work and, as such, had full access to all the data in the study and takes responsibility for the integrity of the data and the accuracy of the data analysis. All authors were involved in the writing of the manuscript and approved the final version in this article.

## Figures and Tables

**Table 1:** Clinical characteristics of study participants

*N*	173
Age (year)	65.0 (10.0)
Sex (male/female)	89/84
Body mass index (kg/m^2^)	24.1 (4.1)
Hemoglobin A1c (%)	7.3 (1.2)
Creatinine (µmol/L)	64.0 (18.6)
eGFR (mL/min/1.73 m^2^)	78.9 (21.0)
Uric acid (µmol/L)	320.1 (89.4)
UAE (mg/g creatinine)	156.4 (491.4)
Triglycerides (mg/dl)	14.1 (88)
Logarithm of triglycerides	2.0 (0.2)
Urinary pH	6.0 (0.6)
Insulin treatment (−/+)	143/40
Antihypertensive drug treatment (−/+)	87/86
Total energy intake (kcal)	1,877.9 (617.1)
Carbohydrate intake (g)	253.3 (79.7)
Protein intake (g)	69.8 (26.5)
Fat intake (g)	56.1 (27.2)
Phosphorus intake (mg/day)	1,060.0 (399.4)
Potassium intake (mg/day)	2,549.0 (1,068.7)
Calcium intake (mg/day)	547.7 (251.4)
Magnesium intake (mg/day)	266.9 (100.2)
Fruit and vegetable intake (g)	265.4 (222.0)
Potential renal acid load score (mEq/day)	5.8 (13.6)

Data are expressed as mean ± SD or absolute number. eGFR, estimated glomerular filtration rate; UAE, urinary albumin excretion.

**Table 2:** Relationship between urinary pH and potential renal acid load score or the other variables

	*r*	*p*
Age	−0.01	0.877
BMI	−0.02	0.825
Hemoglobin A1c	−0.08	0.325
Creatinine	−0.18	0.016
Urinary albumin excretion	−0.14	0.067
Uric acid	−0.16	0.041
Logarithm of triglycerides	−0.11	0.146
Total energy intake	0.03	0.684
Carbohydrate intake	0.07	0.386
Protein intake	0.02	0.789
Fat intake	−0.05	0.533
Fruit and vegetable intake	0.15	0.045
Potential renal acid load	−0.24	0.002

The relationship between urinary pH and potential renal acid load score or the other variables was examined by Spearman’s rank correlation coefficient.

**Table 3:** Multiple regression analysis on urinary pH

	Standardized regression coefficient	*p*
Age	0.05	0.552
Male	0.15	0.125
Body mass index	0.00	0.960
Creatinine	−0.23	0.035
Uric acid	0.00	0.968
Hemoglobin A1c	−0.09	0.265
Logarithm of triglycerides	−0.10	0.240
Fruit and vegetable intake	−0.05	0.598
Potential renal acid load	−0.21	0.036

## Abbreviations

AUC: area under the ROC curve
BMI: body mass index
DHQ: self-administered diet history questionnaire
eGFR: estimated glomerular filtration rate
NNSJ: National Nutrition Survey of Japan
PRAL: potential renal acid load
ROC: receiver operator characteristic
